# IRF1 regulates self-renewal and stress responsiveness to support hematopoietic stem cell maintenance

**DOI:** 10.1126/sciadv.adg5391

**Published:** 2023-10-27

**Authors:** Alexandra J. S. Rundberg Nilsson, Hongxu Xian, Shabnam Shalapour, Jörg Cammenga, Michael Karin

**Affiliations:** ^1^ Laboratory of Gene Regulation and Signal Transduction, Department of Pharmacology, School of Medicine, University of California San Diego, La Jolla, CA, USA.; ^2^ Division of Molecular Medicine and Gene Therapy, Institution for Laboratory Medicine, Medical Faculty, Lund University, Lund, Sweden.; ^3^ Lund Stem Cell Center, Medical Faculty, Lund University, Lund, Sweden.; ^4^ Department of Cancer Biology, The University of Texas MD Anderson Cancer Center, Houston, TX, USA.

## Abstract

Hematopoietic stem cells (HSCs) are tightly controlled to maintain a balance between blood cell production and self-renewal. While inflammation-related signaling is a critical regulator of HSC activity, the underlying mechanisms and the precise functions of specific factors under steady-state and stress conditions remain incompletely understood. We investigated the role of interferon regulatory factor 1 (IRF1), a transcription factor that is affected by multiple inflammatory stimuli, in HSC regulation. Our findings demonstrate that the loss of IRF1 from mouse HSCs significantly impairs self-renewal, increases stress-induced proliferation, and confers resistance to apoptosis. In addition, given the frequent abnormal expression of *IRF1* in leukemia, we explored the potential of *IRF1* expression level as a stratification marker for human acute myeloid leukemia. We show that *IRF1*-based stratification identifies distinct cancer-related signatures in patient subgroups. These findings establish IRF1 as a pivotal HSC controller and provide previously unknown insights into HSC regulation, with potential implications to IRF1 functions in the context of leukemia.

## Teaser

IRF1 controls essential murine HSC functions and provides a way to stratify human AML.

## INTRODUCTION

Hematopoietic stem cells (HSCs) reside at the apex of the hematopoietic hierarchy and ensure life-long blood cell production through their mainly quiescent nature, multilineage differentiation potential, and self-renewal ability (*1*). Inflammation-related signaling pathways control mobilization, proliferation, and differentiation of adult HSCs, thus playing pivotal roles in the regulation of their activity (*2*–*10*). Proper response to inflammation is indispensable for maintaining blood cell homeostasis and the ability to mount appropriate responses to infections and injuries. Dysregulation of these processes can result in leukemia, aging-related HSC impairment, imbalanced blood cell production, and unresolved chronic inflammation. Comprehensive understanding of the roles of specific inflammatory factors under steady-state and stress conditions is crucial to the deciphering of normal and abnormal HSC regulation. Such knowledge can contribute to the development of novel therapeutic interventions against blood disorders.

Interferon (IFN) regulatory factor 1 (IRF1) is a transcription factor (TF) with a central role in innate and adaptive immune responses (*11*). Previous studies have primarily studied IRF1 in mature blood cells under inflammatory contexts, highlighting its involvement in a multitude of cellular processes, including development, immune cell function, pattern recognition receptor signaling, inflammasome activation, proliferation, apoptosis, lipid metabolism, protein degradation, DNA damage, and oncogenesis (*12*–*18*). Various inflammation-related pathways, including tumor necrosis factor (TNF), retinoic acid inducible gene-I–like receptor, Toll-like receptor, and IFN signaling, induce IRF1 expression and activity (*12*). Activated IRF1 binds to IFN-stimulated response elements (ISREs) or IRF-binding elements (IRF-Es) at target gene promoters to activate or repress transcription (*19*, *20*). In addition, IRF1 interacts with the histone acetyltransferases (HATs) E1A binding protein P300 and cyclic adenosine monophosphate response element–binding protein–binding protein (CBP) to regulate gene expression through epigenetic mechanisms (*16*, *21*).

IRF1 has been tied to B and T lymphocyte and myeloid cell development and function (*22*–*29*). *Irf1* transcript levels increase during myeloid differentiation and in response to external stimuli, including viral infections, lipopolysaccharide (LPS), type I and type II IFNs, interleukin-1 (IL-1), IL-12, and granulocyte colony-stimulating factor (*12*, *30*–*37*). IRF1-deficient mice exhibit altered immune cell populations, with decreased numbers of peripheral blood (PB) CD8^+^ T and natural killer (NK) cells, concomitant with increased numbers of CD4^+^ T cells (*28*, *29*), and unaltered levels of red blood cells (RBCs) (*38*). In addition, *Irf1^−/−^* mice display increased frequencies of granulocytic precursors with impaired granulocytic development in the bone marrow (BM) (*38*). However, the role of IRF1 in hematopoietic stem and progenitor cells remains largely unexplored. Notably, HSCs are regulated through different mechanisms than more mature blood cells, including their responses to inflammatory stimuli (*7*, *8*). Deciphering the distinct functions and regulation of IRF1 in various cell types is thus important for a comprehensive understanding of the hematopoietic system.

IRF1 has been implicated in the pathogenesis of acute myeloid leukemia (AML) and myelodysplastic syndrome (MDS) with aberrations of chromosome 5, where the *IRF1* gene is located (*39*). Notably, specific *IRF1* gene mutations have also been observed (*40*). While the exact underlying mechanisms are not fully understood, it was suggested that IRF1 deficiency may lead to inefficient induction of apoptosis in cancer cells (*41*). Although IRF1 deficiency alone typically does not induce cancer, it exacerbates cancer risk and increases the mutation rate in mice carrying the c-Ha-Ras oncogene or that are p53 deficient (*41*). Conversely, certain AML subtypes and the human leukemic TF-1 erythroblast cell line display increased *IRF1* expression (*12*, *42*). Moreover, the IRF1-responsive NOD-, LRR-, and pyrin domain–containing protein 3 (NLRP3) inflammasome (*13*) functions as a driver of MDS (*43*) and mediates glucocorticoid resistance (*44*). Collectively, these studies suggest a potential role of IRF1 in hematological malignancies. Given the limited understanding of IRF1’s involvement in HSC regulation, we undertook this study to elucidate its role in murine HSCs and determine whether IRF1 deficiency in HSCs bears relevance to IRF1 deficiency in human AML.

## RESULTS

### *Irf1^−/−^* mice exhibit altered PB and BM parameters

**Fig. 1.**
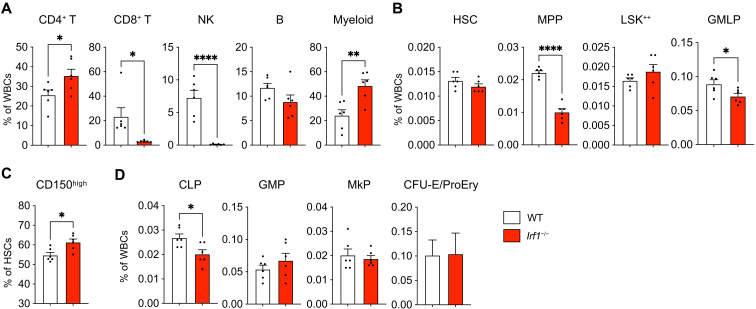
*Irf1*^−/−^ mice show alterations in the composition of PB and BM compartments. (**A**) PB cell distribution within the white blood cell (WBC) fraction. (**B**) LSK subpopulation frequencies within BM WBCs. (**C**) Fraction of HSCs with high CD150 expression. (**D**) Lineage-committed progenitor frequencies within the BM. WT, *n* = 6; *Irf1*^−/−^, *n* = 6. Error bars represent +SEM. *P* values were calculated by two-tailed Student’s *t* test. *P < 0.05, **P < 0.01, and ****P < 0.0001. Littermate WT and *Irf1*^−/−^ mice were used.

To investigate the impact of IRF1 ablation on the hematopoietic system, we analyzed the composition of PB and BM subsets in primary whole-body *Irf1^−/−^* mice compared to wild-type (WT) controls (using the gating strategy outlined in fig. S1A-C). Consistent with previous studies (*38*), *Irf1^−/−^* PB showed prominent reductions in CD8^+^ T and NK cell frequencies, along with increased CD4^+^ T cell frequencies (Fig. 1A). In addition, we observed an expanded myeloid fraction, primarily due to increased neutrophil abundance (fig. S2A). Moreover, *Irf1^−/−^* mice exhibited a trend toward reduced PB B cells. BM analysis showed unaltered frequencies of HSCs [Lineage^−^Sca1^+^cKit^+^ (LSK) CD150^+^ CD48^−^] and LSK CD150^+^ CD48^+^ cells [LSK^++^, also referred to as HPC-2 (*45*)], along with significantly decreased frequencies of multipotent progenitors (MPPs; LSK CD150^−^ CD48^−^) and granulocyte-monocyte lymphoid progenitors [GMLPs, also referred to as HPC-1 (*45*), LSK CD150^−^ CD48^+^; Fig. 1B]. Notably, *Irf1*^−/−^ HSCs exhibited elevated CD150 expression (Fig. 1C), a feature associated with myeloid-skewed, functionally declined, aged HSCs (*46*). Analysis of downstream intermediate and lineage-committed progenitors revealed significantly reduced levels of common lymphoid progenitors (CLPs), while no differences were detected among pre–granulocyte-macrophage progenitors (PreGMs), granulocyte-macrophage progenitors (GMPs), pre–megakaryocytic-erythroid progenitors, pre–colony-forming-unit-erythroid (PreCFU-E) precursors, megakaryocytic progenitors (MkPs), or colony-forming-unit-erythroid/proerythrocyte precursors (CFU-E/ProEry; Fig. 1D and fig. S2B). WT and *Irf1*^−/−^ mice displayed similar total BM counts (fig. S2C). Collectively, these results demonstrate substantial phenotypical deficiencies in PB and BM lymphoid compartments of *Irf1^−/−^* mice, as well as in primitive multipotent BM progenitors.

### IRF1 regulates HSC stress responsiveness

**Fig. 2.**
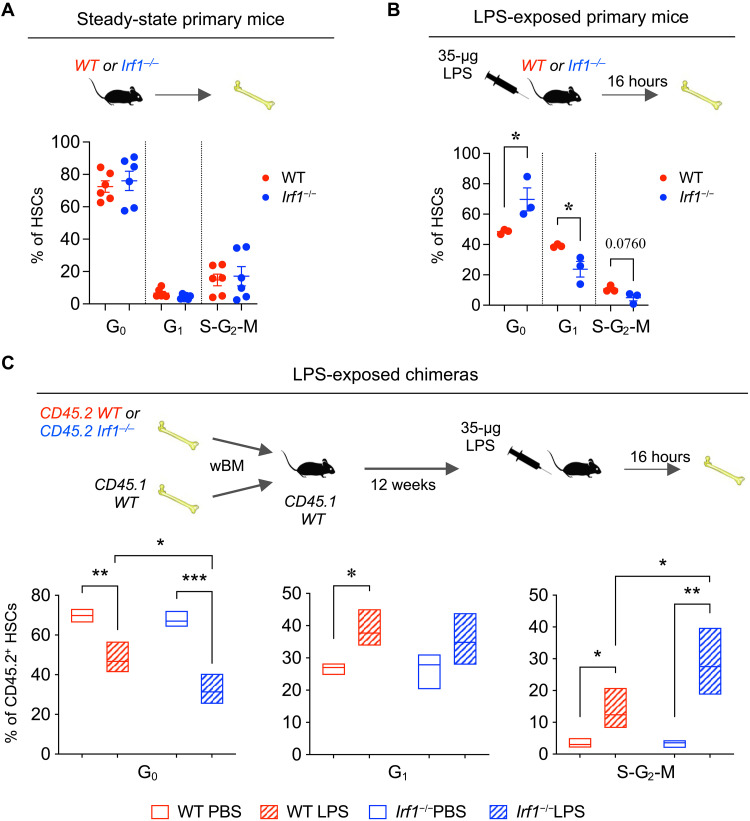
*Irf1*^−/−^ HSCs exhibit an inherently elevated response to LPS. (**A**) HSC cell cycle distribution in steady-state WT and *Irf1*^−/−^ primary mice. WT, *n* = 5; *Irf1*^−/−^, *n* = 5. (**B**) HSC cell cycle distribution in LPS-exposed WT and *Irf1*^−/−^ primary mice. WT, *n* = 3; *Irf1*^−/−^, *n* = 3. Error bars represent ±SEM. (**C**) Donor WT and *Irf1*^−/−^ CD45.2^+^ HSC cell cycle distribution in WT:WT and WT:*Irf1*^−/−^ chimeric mice, respectively. WT phosphate-buffered saline (PBS), *n* = 3; WT LPS, *n* = 5; *Irf1*^−/−^ PBS, *n* = 4; *Irf1*^−/−^ LPS, *n* = 3. Box plots show floating bars (minimum to maximum) with mean line. wBM, whole BM. *P* values were calculated by two-tailed Student’s *t* test. *P < 0.05, **P < 0.01, and ***P < 0.001.

We next evaluated the functionality of *Irf1^−/−^* HSCs. Given IRF1’s role as a negative regulator of proliferation and tumor suppressor in various cell types (*47*, *48*), we examined whether the absence of IRF1 affected HSC cell cycle distribution in primary steady-state mice. Both WT and *Irf1^−/−^* HSCs exhibited predominantly quiescent states, with no significant difference between the two groups (Fig. 2A). However, exposure to prototypical inflammatory stress in the form of LPS, an established IRF1 activator (*13*, *49*), elicited notably diminished HSC activation/proliferation in primary *Irf1^−/−^* mice compared to their WT counterparts (Fig. 2B). To differentiate inherent HSC differences from potential effects of the *Irf1^−/−^* environment, we transplanted WT or *Irf1^−/−^* BM alongside competitive WT BM into WT recipients (Fig. 2C and fig. S3A). When exposed to LPS in this setting, *Irf1^−/−^* HSCs exhibited significantly higher cell cycle activity compared to WT HSCs (Fig. 2C). Control groups showed no significant differences in cell cycle activity (Fig. 2C and fig. S3B). While acknowledging potentially differential effects of the transplantation procedure on WT and *Irf1^−/−^* HSCs that could influence their response to inflammatory stimuli, these findings collectively suggest that *Irf1^−/−^* HSCs have inherently enhanced responsiveness to LPS, which is extrinsically suppressed by the *Irf1^−/−^* environment.

### *Irf1^−/−^* HSCs show impaired long-term repopulation capacity

**Fig. 3.**
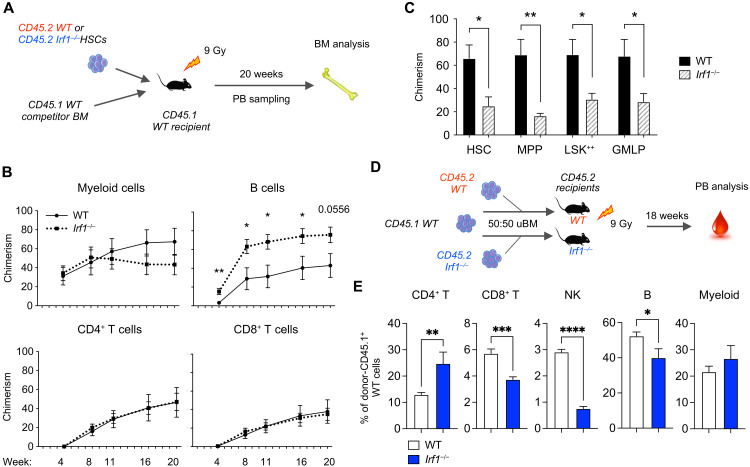
*Irf1*^−/−^ HSCs display reduced repopulation capacity. (**A**) Experimental strategy for competitive transplantations comparing WT and *Irf1*^−/−^ HSCs. Results are depicted in (B and C). (**B**) Donor CD45.2^+^ PB chimerism levels at various time points after competitive HSC transplantation. Error bars represent ±SEM. (**C**) Chimerism levels for immature LSK subpopulations in the BM at 1° transplantation end point. Error bars indicate +SEM. WT, *n* = 6; *Irf1*^−/−^, *n* = 6. (**D**) Experimental strategy for the investigation of the environmental impact on WT-derived blood cell output. Results are depicted in (E). (**E**) CD45.1^+^ WT donor cell distribution of PB cell compartments within WT (white) and *Irf1*^−/−^ (blue) hosts. Error bars indicate +SEM. Recipients: WT, *n* = 8; *Irf1*^−/−^, *n* = 6. *P* values were calculated by two-tailed Student’s *t* test. *P < 0.05, **P < 0.01, ***P < 0.001, and ****P < 0.0001. Gy, gray.

To specifically investigate the cell-autonomous effects of IRF1 loss on HSC long-term self-renewal and reconstitution ability, we conducted competitive HSC transplantation experiments (Fig. 3A). These experiments revealed comparable chimerism levels in the PB T cell and myeloid compartments of WT and *Irf1^−/−^* HSCs at early time points after transplantation (4 and 8 weeks; Fig. 3B). However, *Irf1^−/−^* B cell chimerism was considerably higher at all evaluated posttransplantation time points, resulting in a prominent B cell–biased distribution (Fig. 3B and fig. S4A). Beginning at 11 weeks after transplantation, we observed a trend toward reduced levels of myeloid cells, which have a high turnover rate, while T cells, which display much slower turnover, remained unchanged (Fig. 3B). This pattern is consistent with HSC exhaustion. At the end point, all evaluated BM subsets, including HSCs, exhibited considerably reduced *Irf1^−/−^* chimerism levels (Fig. 3C). Secondary competitive transplantation of repurified HSCs confirmed a reduced long-term reconstitution capacity of *Irf1^−/−^* HSCs (fig. S4B). These results provide evidence for an impaired self-renewal capacity of *Irf1^−/−^* HSCs.

Given the differences in PB cell distribution between primary *Irf1^−/−^* mice (Fig. 1A) and the *Irf1^−/−^* donor-derived output in transplanted mice (fig. S4A), we sought to evaluate the contribution from the *Irf1^−/−^* microenvironment to the observed PB perturbations. For this purpose, we generated WT;WT and WT;*Irf1^−/−^* BM chimeras in WT and *Irf1^−/−^* recipients, respectively (Fig. 3D). WT cells in the *Irf1^−/−^* environment displayed extensive differences in PB lineage distribution compared to WT cells in the WT environment (Fig. 3E). These changes replicated the alterations observed in primary *Irf1^−/−^* mice, including an augmented CD4^+^ T cell distribution and reductions in CD8^+^ T and NK cells, suggesting that the *Irf1^−/−^* environment extrinsically affects these lineages. Although these findings cannot fully differentiate between the influence of the hematopoietic and nonhematopoietic *Irf1^−/−^* microenvironments, previous studies have demonstrated that *Irf1^−/−^* immature thymocytes impede the production of CD8^+^ T cells in *Irf1^−/−^* mice (*50*). Notably, the *Irf1^−/−^* environment significantly reduced WT B cell frequencies (Fig. 3E). This relative reduction of WT B cells was not caused by an expansion of endogenous/competitive *Irf1^−/−^* B cells in the same mouse (fig. S4C), suggesting that transplantation-associated expansion of *Irf1*^−/−^ B cells (Fig. 3B and fig. S4A) requires a WT environment. Together, these findings imply that the IRF1-deficient environment extrinsically impedes CD8^+^ T, NK, and B lymphoid cell production.

### *Irf1^−/−^* HSCs display an altered transcriptomic profile

**Fig. 4.**
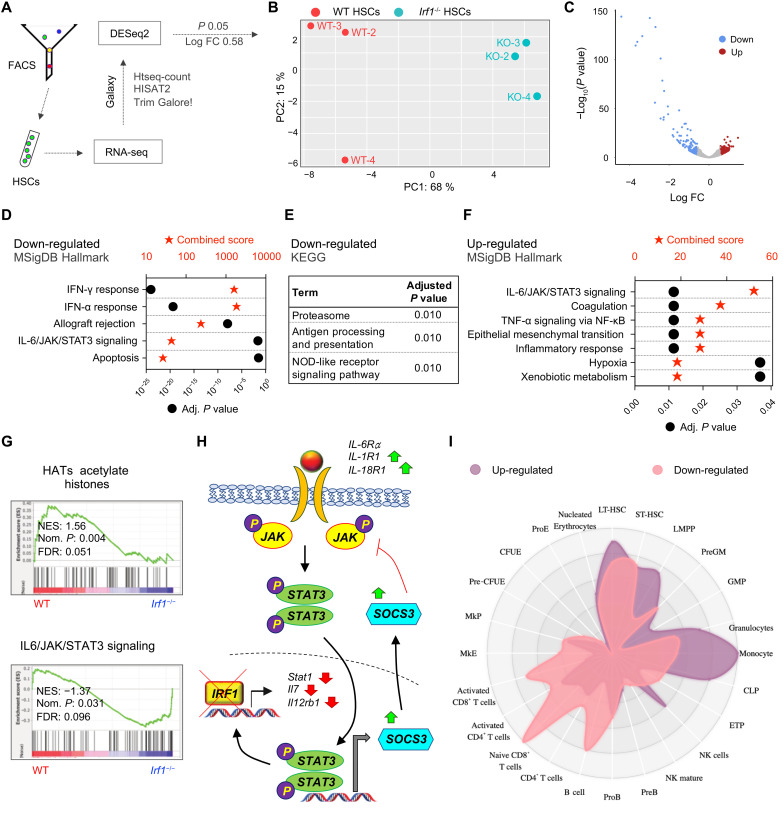
*Irf1*^−/−^ HSCs display a transcriptional profile consistent with reduced function. (**A**) Experimental strategy for gene expression analysis. (**B**) Principal components analysis (PCA) plot showing the relationship between the samples. (**C**) Volcano plot indicating differentially expressed genes (DEGs) between WT and *Irf1*^−/−^ HSCs. Red and blue dots represent significant DEGs with log FC > 0.58 and significance threshold <0.05. Enrichment of (**D**) MSigDB Hallmark and (**E**) Kyoto Encyclopedia of Genes and Genomes (KEGG) gene sets within down-regulated genes in *Irf1*^−/−^ HSCs. (**F**) Enrichment of MSigDB Hallmark gene sets within up-regulated genes in *Irf1*^−/−^ HSCs. (**G**) Conventional GSEA on WT and *Irf1*^−/−^ HSC gene expression profiles for the depicted gene sets. (**H**) Significantly up- (green) and down-regulated (red) IL-6/JAK/STAT3 pathway components in *Irf1*^−/−^ HSCs. (**I**) Lineage enrichment analysis of significantly up- and down-regulated genes in *Irf1*^−/−^ HSCs. FACS, fluorescence-activated cell sorting. NF-κB, nuclear factor κB; FDR, false discovery rate.

We next performed bulk RNA sequencing (RNA-seq) of HSCs from WT and *Irf1^−/−^* mice to uncover the transcriptomic changes underlying the functional alterations observed in *Irf1^−/−^* HSCs (Fig. 4A). Differential gene expression analysis using DESeq2 [adjusted *P* < 0.05, log fold change (FC) > 0.58] revealed 169 up-regulated and 134 down-regulated genes in *Irf1^−/−^* HSCs compared to WT HSCs (Fig. 4, B and C, and tables S1 and S2). Ingenuity pathway analysis (IPA) (*51*) predicted IRF1, along with the IRF1 direct targets signal transducer and activator of transcription 1 (STAT1) and tripartite motif-containing 24 (TRIM24) (*18*), as the top three transcriptional regulators of the down-regulated genes (table S3). Consistent with the role of IRF1 in other cell types, *Irf1^−/−^* HSCs showed suppression of IFN-α and IFN-γ responses (*12*, *20*, *26*, *52*), apoptosis (*12*, *53*–*55*), antigen processing and presentation (*56*–*59*), and proteasome components (Fig. 4, D and E, and tables S4 and S5) (*60*). In addition, *Irf1^−/−^* HSCs exhibited diminished unfolded protein response (UPR) and down-regulation of components associated with key cellular integrity functions, such as the spliceosome, multiple DNA repair pathways, and epigenetic regulation (Fig. 4G and fig. S5, A and B). Aligning with IRF1’s previously reported interaction with the HATs p300/CBP (*16*, *21*), *Irf1*^−/−^ HSCs displayed down-regulation of genes near p300 DNA binding sites (fig. S5C and table S6). Despite no significant differences in HSC cell cycle status in primary *Irf1^−/−^* mice (Fig. 2A), the RNA-seq analysis suggested a decreased cell proliferation signature in *Irf1^−/−^* HSCs (fig. S5D). This finding may be linked to the observed reduction in LPS-induced HSC proliferation in primary *Irf1^−/−^* mice (Fig. 2B).

Notably, both up-regulated and down-regulated genes were enriched for the IL-6/Janus kinase (JAK)/STAT3 signaling gene set (Fig. 4, D and F, and tables S4 and S7). Consistent with the IRF1 deficiency, most of the down-regulated genes were downstream of IRF1 in the IL-6/JAK/STAT3 pathway (Fig. 4H) and contained IRF1 binding sites (*18*). In contrast, the up-regulated genes, including IL-6 receptor, did not have IRF1 binding sites and were situated upstream to the IL-6/JAK/STAT3 module or were direct STAT3 targets. Gene set enrichment analysis (GSEA) revealed a significant overall enrichment of the IL-6/JAK/STAT3 pathway in *Irf1^−/−^* HSCs (Fig. 4G), suggesting a lesser contribution from downstream IRF1 target genes to this gene set. In addition, transcriptomic analysis revealed significant up-regulation of other inflammatory pathways in *Irf1^−/−^* HSCs, including TNF signaling (Fig. 4F). These results underscore the intricacy of inflammatory networks in *Irf1^−/−^* HSCs, where gene sets that rely heavily on downstream IRF1 targets are repressed, while several others are up-regulated.

Considering the increased CD150 expression on *Irf1^−/−^* HSCs and the challenge in functionally determining HSC lineage priming in primary and transplanted mice due to the strong downstream influences, we also examined enrichment of cell type–specific gene sets in WT and *Irf1^−/−^* HSCs. *Irf1^−/−^* HSCs up-regulated both lymphoid (CLP) and myeloid (PreGM) genes (*61*), while down-regulating megakaryocytic (MkP) and erythroid (PreCFU-E) genes (fig. S5E and table S8) (*61*). Cell type enrichment analysis revealed that the 169 significantly up-regulated genes in *Irf1^−/−^* HSCs were associated with multipotent cell types, granulocytes, monocytes, and CLPs, while the 134 significantly down-regulated genes were associated with B and T cells, as well as immature multipotent cells (Fig. 4I). Moreover, *Irf1^−/−^* HSCs displayed enrichment of an HSC-specific gene set (fig. S5F). Nonetheless, enrichment of HSC gene sets, often derived from comparisons between HSCs and more differentiated progenitors, does not necessarily indicate improved function, as shown by observations between aged WT HSCs versus their younger counterparts (*61*). To gain a more comprehensive insight, we therefore conducted GSEA using gene sets derived from aged HSCs (*61*) and *Stat1*^−/−^ HSCs (*62*), acting as proxies for HSCs with compromised function. These analyses indicated reduced stem cell function in *Irf1^−/−^* HSCs (fig. S5F). Collectively, these findings suggest a marked deviation in the transcriptional profile of *Irf1^−/−^* HSCs consistent with a compromised HSC function.

It is important to acknowledge that these transcriptional findings were obtained in the context of a whole-body IRF1 deficiency, where the influence of nonhematopoietic and hematopoietic *Irf1^−/−^* cells on HSCs cannot be excluded. Nevertheless, our findings provide valuable insights into IRF1-regulated genes in HSCs that were not subject to transplantation stress, revealing reduced functionality, blunted apoptosis, and altered immune signaling in *Irf1^−/−^* HSCs.

### IRF1 controls apoptosis, protein ubiquitination, and major histocompatibility complex class II expression

**Fig. 5.**
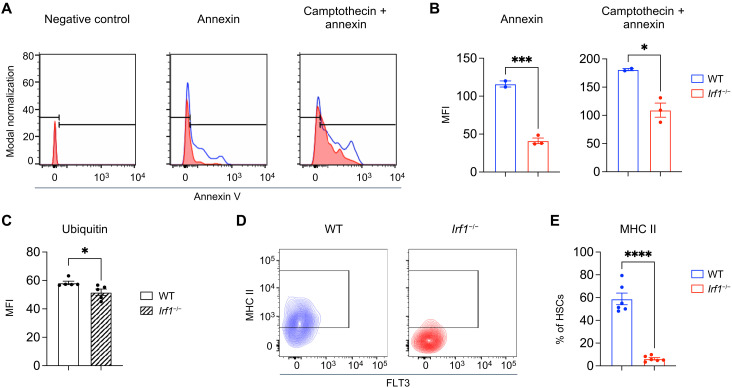
*Irf1*^−/−^ HSCs show enhanced survival, reduced protein ubiquitylation, and diminished cell surface MHC class II expression. (**A**) Representative histograms of annexin V in WT (blue) and *Irf1*^−/−^ (red) HSCs as a measurement of apoptosis. (**B**) Quantification of mean fluorescence intensity (MFI) in (A). (**C**) Quantification of ubiquitin in WT and *Irf1*^−/−^ HSCs. (**D**) Representative plots visualizing the expression of MHC class II molecules on the surface of WT and *Irf1*^−/−^ HSCs. (**E**) Quantification of the frequency of MHC class II–positive HSCs in WT and *Irf1*^−/−^ mice. Error bars represent ±SEM. *P* values were calculated by two-tailed Student’s *t* test. *P < 0.05, ***P < 0.001, and ****P < 0.0001.

The above RNA-seq analysis was consistent with previous studies reporting blunted apoptosis in IRF1-deficient cells (*12*, *53*–*55*). Reduced apoptosis may partly compensate for the impaired functionality of *Irf1^−/−^* HSCs in primary mice and could contribute to their maintenance at early time points after transplantation. To experimentally validate this transcriptional finding, we measured basal apoptosis in HSCs using annexin V staining and flow cytometry. Consistent with the transcriptomic analysis, *Irf1^−/−^* HSCs displayed reduced annexin V staining compared to WT controls (Fig. 5, A and B, and fig. S6A). In addition, when exposed to camptothecin, an apoptosis-inducing DNA-damaging agent, *Irf1^−/−^* HSCs continued to exhibit lower levels of apoptosis relative to WT HSCs, further indicating that IRF1 deficiency confers apoptosis resistance in HSCs.

We also observed a reduction in UPR-associated genes and transcripts linked to the proteasome and DNA repair systems (Fig. 4E and fig. S5A). Dysregulation of these systems affects HSC function and promotes transformation and malignancy (*63*). To functionally address the reduced proteasome and UPR signaling, we quantified the levels of ubiquitinated proteins in WT and *Irf1^−/−^* HSCs. Our analysis revealed significantly lower amounts of ubiquitin in *Irf1^−/−^* HSCs (Fig. 5C), providing further support for the dysregulation of this pathway.

Moreover, our transcriptional profiling demonstrated a notable reduction in the expression of genes involved in antigen presentation and processing in *Irf1^−/−^* HSCs (Fig. 4E and fig. S6B). These included several major histocompatibility complex (MHC) class II genes, such as *H2-Eb1*, *H2-Ab1*, *H2-Aa*, *H2-Q6*, and *H2-DMa*, and known controllers of MHC class II expression, *Ciita* and *Stat1* (fig. S6B and table S1). MHC class II expression was recently linked to HSC functionality (*62*, *64*) and is regulated by IRF1 in other cell types (*56*–*58*). This prompted us to validate MHC class II surface expression in *Irf1^−/−^* HSCs, revealing a notable 10.5-fold reduction in MHC class II levels (Fig. 5, D and E), which may have functional consequences for *Irf1^−/−^* HSCs. Collectively, these findings establish IRF1 as a crucial regulator of HSC homeostasis and function.

### *IRF1* expression marks distinct AML patient subgroups

**Fig. 6.**
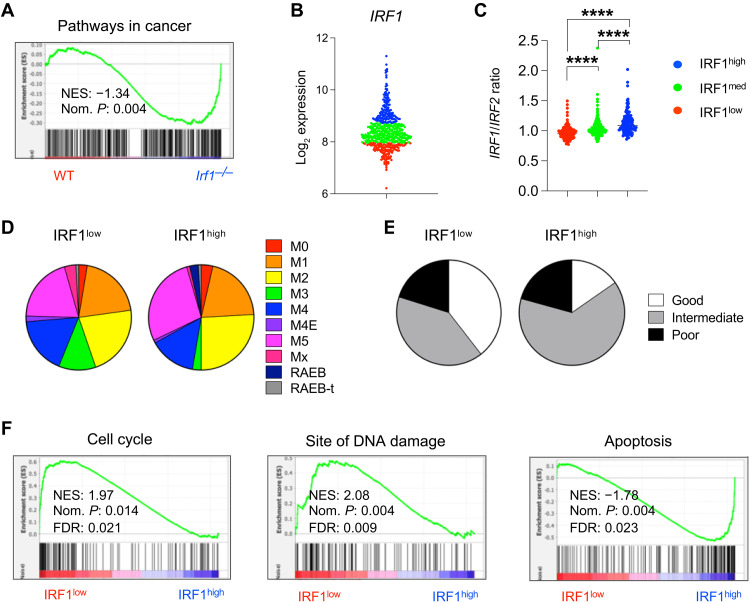
AML patient stratification based on *IRF1* expression reveals distinct cancer-associated features. (**A**) Enrichment of the Pathways in cancer gene set between WT and *Irf1*^−/−^ murine HSCs. (**B**) *IRF1* expression–based patient stratification of 537 human AML patient samples for the analyses depicted in (C) to (F). (**C**) IRF1/IRF2 ratio in IRF1^low^ (*n* = 135), IRF1^medium^ (*n* = 268), and IRF1^high^ (*n* = 134) AML patient samples. *P* values were calculated by two-tailed Student’s *t* test. ****P < 0.0001. Distribution of (**D**) FAB scores (IRF1^low^, *n* = 114; IRF1^high^, *n* = 111) and (**E**) good, intermediate, and poor cytogenetic risk (IRF1^low^, *n* = 109; IRF1^high^, *n* = 111), within IRF1^low^ and IRF1^high^ AML patient subgroups. (**F**) GSEA between IRF1^low^ and IRF1^high^ patient samples for cell cycle, site of DNA damage, and apoptosis gene sets.

Given the reported dysregulation of IRF1 in myeloid malignancies (*39*, *65*), which may parallel its role in murine HSC function, we explored the potential of using *IRF1*-based stratification to identify distinct subgroups in human AML. Our observations that IRF1 regulates HSC features that are often exploited by cancerous cells, such as self-renewal, survival, and expression of MHC class II, suggest that IRF1 dysregulation may promote leukemogenesis and therapy resistance. In further support of this postulate, we found that *Irf1^−/−^* HSCs displayed enrichment of the “Pathways in cancer” gene set (Fig. 6A).

To investigate *IRF1*-based stratification, we used a previously published dataset of 537 adult human patients with AML (GSE5891). The AML samples were allocated into three groups: IRF1^high^ (the top 25% expressing samples), IRF1^low^ (bottom 25% expressing samples), and IRF1^medium^ for the remaining samples (Fig. 6B). We found that the *IRF1:IRF2* expression ratio, which is reduced in AML compared to healthy BM (*66*), significantly decreased with reduced *IRF1* expression (Fig. 6C). Moreover, consistent with the presence of the *IRF1* gene on chromosome 5, the IRF1^low^ group contained a higher fraction of patients with loss of 5 of 7(q) (fig. S7A). In addition, IRF1^low^ AML enriched for patients with t(8;21) and t(15;17) translocations and had fewer patients with a normal karyotype (NN). The IRF1^high^ group, on the other hand, had fewer patients with chromosome inversion 16. Furthermore, IRF1^low^ and IRF1^high^ samples displayed differences in the French-American-British (FAB) classification of AML (Fig. 6D). While the IRF1^high^ AML group contained more patients with the M5 acute monocytic leukemia subtype, the IRF1^low^ subgroup had more patients with M3 acute promyelocytic leukemia and M4 acute myelomonocytic leukemia. Notably, the IRF1^low^ AML group contained a considerably higher proportion of patients with good cytogenetic risk compared to IRF1^high^ samples (Fig. 6E). These findings indicate that *IRF1*-based stratification provides valuable insight into disease characteristics and clinical outcomes in AML.

Transcriptomic analysis revealed that IRF1^low^ AML exhibited increased expression of genes involved in DNA damage and repair (Fig. 6F and fig. S7B). Moreover, IRF1^low^ AML samples displayed enhanced survival and proliferation, as well as reduced inflammatory signaling and differentiation (fig. S7B). These results imply that *IRF1*-based stratification can be used to identify AML subgroups with distinct underlying cellular mechanisms.

Last, comparison of *IRF1* expression among various human leukemia subclasses (GSE13159) revealed significant down-regulation of *IRF1* in most leukemia subclasses compared to healthy BM (fig. S7C). However, some lymphoid leukemias exhibited significant up-regulation of *IRF1*, and *IRF1* expression varied substantially between patients within all leukemia subclasses. While further studies are required to decipher the role of IRF1 in leukemia and the relationship between *IRF1* expression and clinical features, such as overall survival and therapy response, our results suggest the potential of *IRF1*-based patient stratification for identification of AML subgroups with distinct disease features.

## DISCUSSION

IRF1 has been extensively studied in mature blood cells, particularly in the context of inflammation, and was shown to control various cellular processes, including apoptosis, proliferation, and expression of MHC class I and II molecules. However, its role in the HSC compartment was barely explored. Nevertheless, several inflammatory mediators that signal via IRF1 affect HSC activity (*2*, *4*, *6*, *7*), highlighting its central position in intracellular signaling and suggest a potential involvement in HSC regulation. In this study, we demonstrate a critical role for IRF1 in regulating essential HSC features, including self-renewal, survival, stress-induced proliferation, and expression of immunogenic cell surface proteins, such as MHC class II (summarized in fig. S8). Our findings reveal both shared and distinct functions of IRF1 between HSCs and other cell types, underscoring the complexity of IRF1-mediated regulation in various cellular contexts. These insights into HSC regulation expand our understanding of the intricate mechanisms governing hematopoiesis and provide previously unexplored avenues for investigating the role of IRF1 in HSC biology. Some of these findings may also be relevant to human AML with dysregulated *IRF1* expression.

Although phenotypical assessment of primary *Irf1^−/−^* mouse BM did not reveal overt functional deficiencies of *Irf1^−/−^* HSCs, competitive HSC transplantation assays demonstrated that *Irf1^−/−^* HSCs had significantly reduced reconstitution capacity. Transcriptomic analysis corroborated these impairments, unveiling dysregulated inflammatory signaling and enrichment of functionally impaired HSC signatures in the absence of IRF1. In addition, we observed defects in histone modification pathways, including HATs, suggesting that IRF1 may regulate HSC functionality through epigenetic mechanisms. Notably, the related HATs CBP and P300, which interact directly with IRF1 in human glioblastoma and colon cancer cell lines (*16*, *21*), are essential for HSC and embryonic stem cell self-renewal and differentiation (*67*, *68*). We showed that *Irf1*^−/−^ HSCs down-regulate genes containing P300 binding sites, suggesting that IRF1 regulates P300/CBP activity also in HSCs. Furthermore, *Irf1^−/−^* HSCs displayed reduced apoptosis and UPR, as well as down-regulation of multiple DNA repair pathways and proteasome components, indicating compromised cellular integrity and homeostasis over time or in response to stress, as observed after transplantation. These findings align with previous studies demonstrating an IRF1 requirement for DNA damage–induced cell death (*69*) and that IRF1 deficiency confers apoptosis resistance in various cancer cells (*53*–*55*). In addition to apoptosis, IRF1 has been implicated in necroptosis (PANoptosis) and pyroptosis (*54*).

The role of MHC class II in HSCs remains a subject of debate (*62*, *64*). A recent study showed that WT HSCs with low MHC class II expression exhibit higher reconstitution potential but are more susceptible to apoptosis and display increased proliferation upon exposure to 5-fluoracil and polyinosinic:polycytidylic acid compared to WT HSCs with high MHC class II expression. Consistent with these findings, we observed that *Irf1*^−/−^ HSCs, which exhibited substantially decreased MHC class II surface expression, exhibited an inherently increased proliferation response to LPS within a WT environment. However, contradictory results, such as reduced apoptosis and diminished reconstitution potential in *Irf1*^−/−^ HSCs, suggest a more intricate relationship between MHC class II, IRF1, and HSC functionality. Similar conclusions can be drawn from investigations of HSCs deficient for the IRF1-target gene *Stat1* (*18*), which also exhibited lower MHC class II amounts and decreased repopulation capacity (*62*).

While *Irf1*^−/−^ HSCs displayed an enhanced LPS-induced proliferation capacity in BM chimeras in WT hosts, the contrary was observed in primary *Irf1*^−/−^ mice, suggesting a suppressive effect of the *Irf1*^−/−^ environment. This dichotomy underscores the complex interplay between intrinsic HSC properties and external cues provided by the *Irf1*^−/−^ milieu. Considering the aforementioned findings and the established role of IRF1 in the production of molecules recognized for their HSC activating properties, it is plausible that the reduced LPS-induced HSC proliferation observed in primary *Irf1*^−/−^ mice can be attributed, at least in part, to diminished indirect signaling via such molecules from *Irf1*^−/−^ non-HSCs. Although not specifically explored within the scope of this study, it is conceivable that *Irf1^−/−^* HSCs have an inherent predisposition toward heightened proliferation in response to direct sensing of diverse inflammatory mediators. This notion is supported by the observed elevated expression of cell surface receptors for IL-6, IL-1, and IL-18 in *Irf1^−/−^* HSCs, implying an augmented responsiveness to these cytokines (at least upstream of IRF1 in these signaling pathways). Reverse transplantations uncovered that the *Irf1*^−/−^ environment also repressed CD8^+^ T, NK, and B lymphoid cell production. The transplantation of *Irf1^−/−^* cells into WT recipients, but not *Irf1^−/−^* recipients, triggered an expansion of *Irf1^−/−^* B cells. This expansion may be linked to previous observations of intrinsically regulated *Irf1^−/−^* B cell expansion in response to infectious stress (*22*–*24*) or to the cell-autonomous expansion of B cells lacking MHC class II (*70*). Further investigation is needed to determine the precise contribution of specific hematopoietic and nonhematopoietic cells to these effects.

In this study, we used whole-body knockout (KO) mice to examine the role of IRF1 in HSCs and acknowledge the potential influence of nonhematopoietic cells in the observed phenotypes. Utilization of conditional KO models for IRF1 will be valuable for further investigations, enabling exploration of potential interactions between hematopoietic and nonhematopoietic cells in driving the functional changes in *Irf1^−/−^* HSCs. To exclude the influence from the *Irf1^−/−^* nonhematopoietic environment and investigate the intrinsic effect of IRF1 deficiency on HSC function, we conducted competitive HSC transplantations. Although competitive HSC transplantation is considered the gold standard for assessing HSC function, it should be noted that this approach generates perturbations and may not fully replicate physiologically relevant contexts. Furthermore, this approach does not mitigate the influence of the IRF1-deficient environment during developmental stages before the harvest of HSCs. Nevertheless, our findings highlight the multifaceted involvement of IRF1 in maintaining HSC function. Further investigations are warranted to elucidate the precise mechanisms through which IRF1 regulates these processes and uncover its intricate roles as both a TF and an epigenetic regulator.

Down-regulation of *IRF1* is frequently observed in MDS and AML and increases tumor predisposition and mutation rates in mice when combined with other cancer-promoting mutations (*41*). On the basis of our results in HSCs, we propose that reduced IRF1 may contribute to oncogenesis through various mechanisms, including immune evasion via MHC class II down-regulation, clonal evolution through DNA damage and mutagenesis, and expansion via enhanced proliferation and apoptosis resistance. Increased IRF1 activity may also contribute to oncogenesis through augmented self-renewal. Considering the potential involvement of IRF1 in leukemia, we conducted *IRF1* expression level–based stratification of human AML. Leukemia patient stratification is important for personalized medicine and can be used to define specific pathological processes, improve treatment prognosis and responsiveness, and tailor suitable therapy. It should, however, be noted that some of the comparisons between our results in mouse HSCs and human AML are challenging because of a whole-body IRF1 KO environment versus high and low *IRF1* gene expression in hematopoietic cells, steady-state murine conditions versus a full-blown perturbed leukemic context, and differences in the specific cell type(s) being investigated. Nevertheless, our findings suggest that *IRF1* expression can be used to identify subclasses of leukemia with diverse underlying mechanisms and clinical characteristics, including cancer-associated gene signatures, differences in cell proliferation, survival, DNA damage responses, and differentiation, as well as in karyotype and cytogenetic risk distribution. Moreover, our observation that *IRF1* expression differs substantially between patients within various leukemia subclasses supports the use of IRF1 as a broad leukemia stratification marker.

## MATERIALS AND METHODS

### Mice

Age- and gender-matched *Irf1*^−/−^ (B6.129S2-Irf1^tm1Mak^/J), C57Bl/6, and B6.SJL-Ptprx^a^Pepc^b^/Boy mice were used throughout the study. The mice were maintained at the University of California San Diego Leichtag building vivarium. All procedures were conducted in accordance with ethical permit S00218 approved by the Institutional Animal Care and Use Committee. Three-month-old male mice were used for the primary phenotyping analyses. Four-month-old male mice were used for steady-state cell cycle analysis. Three-month-old female mice were used for LPS-induced cell cycle examination of primary mice. Eight-week-old male donors and 14-week-old female recipients were used for cell cycle analysis in chimeras. Two-month-old female donors and recipients were used for competitive HSC transplantation experiments. Three-month-old female recipients, 6-week-old WT and *Irf1*^−/−^ CD45.2^+^ male donors, and 3-month-old CD45.1^+^ male competitive donors were used for reverse transplantations. Eight-week-old female mice were used for RNA-seq analysis. Five-month-old female mice were used for the apoptosis analysis. Two-month-old female mice were used for the ubiquitin assay. Three-month-old male mice were used for MHC class II expression analysis.

### Isolation and analysis of PB and BM

PB and BM were isolated and analyzed as previously described (*61*, *71*, *72*). Briefly, PB was collected in fluorescence-activated cell sorting (FACS) buffer [phosphate-buffered saline (PBS), 2% fetal bovine serum, and 2 mM EDTA] supplemented with 0.0004% heparin solution (STEMCELL Technologies Inc.). RBCs were removed by incubation in 1% dextran solution in PBS at 37°C for 25 min. The cells in the supernatant were isolated and subjected to room-temperature RBC lysis solution (STEMCELL Technologies Inc.). PB cells were stained with conjugated antibodies targeting CD4, CD8, NK1.1, CD19, CD11b, Gr-1, CD45.1, and CD45.2. BM cells were isolated from tibias, femurs and hip bones that were crushed with a pestle and mortar. Lineage depletion (B220, CD4, CD8, CD11b, Gr-1, and Ter119) or c-kit enrichment was performed using MACS magnetic microbead kits (Miltenyi Biotec) according to the manufacturer’s instructions. BM cells were stained with conjugated antibodies against B220, CD4, CD8, CD11b, Gr-1, Ter119, cKit, Sca1, CD48, CD150, CD45.1, CD45.2, CD105, CD41, CD16/32, and MHC class II. Streptavidin-BV510 was used for biotin identification. Propidium Iodide (1 μg/ml; Molecular Probes) was used to distinguish viability. Flow cytometry analysis and assisted cell sorting were performed using Beckman Coulter CyAn ADP, Becton Dickinson (BD) LSRFortessa X-20, and BD Aria III.

### LPS treatments

Mice were administered 35 μg of LPS (Sigma Aldrich) intraperitoneally and euthanized 16 hours later for downstream analysis. Control mice were administered volume-equivalent 0.9% saline solution.

### Transplantations

Competitive HSC transplantations were performed using 100 Lin^−^Sca-1^+^Kit^+^CD150^+^CD48^−^ HSCs together with 300,000 whole BM (wBM) competitor cells. wBM chimeras were generated with 10 million total donor BM cells. Recipient mice were lethally irradiated with 9 gray and treated prophylactically with sulfamethoxazole and trimethoprim antibiotics for 2 weeks after transplantation in the drinking water. For serial transplantations, 276 donor HSCs were reisolated from each donor and transplanted together with 300,000 wBM competitor cells.

### Cell cycle analysis

Lineage-depleted BM cells were stained with HSC-identifying antibodies before fixation and permeabilization with BD Perm/Wash buffer (BD Pharmingen, Becton, Dickson and Company) at 4°C for 20 min. Cells were subsequently incubated with anti-Ki67 or immunoglobulin G 1 (IgG1) isotype control before being washed and resuspended in 7-aminoactinomycin D (10 μg/ml) diluted in Perm/Wash solution and incubated at 4°C overnight before analysis.

### Apoptosis assay

BD Annexin V: FITC Detection Kit I (BD Pharmingen) was used according to the manufacturer’s instructions. Briefly, lineage-depleted BM cells were stained with HSC-defining antibodies before being washed and incubated with camptothecin at a final concentration of 5 μM for 5 hours at 37°C. The samples were then washed with ice-cold FACS buffer and resuspended in 1× annexin binding buffer to which annexin V and propidium iodide were added. The samples were vortexed and incubated in the dark for 15 min at room temperature. Additional annexin binding buffer was then added after which the samples were analyzed within 1 hour.

### Ubiquitin

After lineage depletion, antibody staining, and fixation, cells were incubated with anti–multi-ubiquitin antibody for 30 min at 4°C. The cells were then washed and resuspended with goat anti-mouse IgG Alexa Fluor 488 (Invitrogen) before analysis.

### RNA-seq and analysis

About 1600 HSCs were sorted from each WT and *Irf1*^−/−^ mouse into RLT lysis buffer supplemented with (1:100) β-mercaptoethanol. RNA was isolated using the Single Cell RNA Purification Kit (Norgen Biotek Corp.). cDNA generation and amplification were performed using the SMART-Seq v4 Ultra Low Input RNA Kit for sequencing (Takara Bio USA Inc.). Quality check was performed using High Sensitivity D5000 ScreenTape with TapeStation Analysis Software 3.2 (Agilent Technologies Inc.). Sequencing was performed using Nova Seq S4 (run type, PE100; type of library, Nextera XT; 25 million reads per sample; Illumina). Preprocessing and analysis were done using The Galaxy platform (*73*). Trim Galore! was used for quality check and adapter trimming of reads; HISAT2 was used for alignment and annotation, and Htseq-count was used to count aligned reads. Differentially expressed genes were identified using DESeq2. Volcano plots were generated with log FC = 0.58 and significance threshold of 0.05. The *P* value was adjusted for multiple testing with the Benjamini-Hochberg procedure, which controls false discovery rate. Enrichment analyses of up- and down-regulated genes was performed with Enrichr (*74*–*76*) and WEB-based Gene SeT AnaLysis Toolkit (WebGestalt) (*77*). Global gene set enrichment analysis was performed with GSEA software (*78*, *79*). Upstream analysis was done with QIAGEN IPA (https://digitalinsights.qiagen.com/IPA). Cell type enrichment of up- and down-regulated genes was performed with CellRadar (*80*).

### AML patient stratification

Publicly available expression data from GSE6891 containing 537 human AML patient samples (<60 years of age) was used for patient stratification (*81*, *82*). The 134 samples (~25%) with the highest expression of *IRF1* were allocated to the IRF1^high^ group, and the 135 samples (~25%) with the lowest IRF1 expression were allocated to the IRF1^low^ group. The middle expressing samples (*n* = 268) were defined as IRF1^med^. In addition, available data were used to assess karyotype, FAB score, and cytogenetic risk distribution between the groups. Gene expression profiles were used to subject the groups to global GSEA. Publicly available expression data from GSE13159 were used to evaluate the expression of *IRF1* in various leukemia subclasses.

### Statistical analysis

Statistical significance between experimental groups analyzed by flow cytometry was determined by unpaired Student’s *t* tests using Prism 9 (GraphPad software). Statistical details can be found in the figure legends. Division of mice into groups was randomized but not blind, and no statistical methods were used to determine the number of mice for the experiments. Multilineage (T + B + My) donor reconstitution levels below 1% in primary recipients were considered unsuccessful and excluded (*P < 0.05, **P < 0.01, ***P < 0.001, and ****P < 0.0001). Analysis of gene expression data is described above. Detailed information about antibodies, reagents, and instruments can be found in table S9.
