# Supplementary material for: IRF1 regulates self-renewal and stress responsiveness to support hematopoietic stem cell maintenance
Source: Sci Adv. 2023 Oct 27;9(43):eadg5391. doi: 10.1126/sciadv.adg5391 (PMC10610924; doi:10.1126/sciadv.adg5391)
Supplement: Supplementary file 1 — Figs. S1 to S8 Tables S4, S5, S6, S7 and S9 Legends for tables S1, S2, S3 and S8 [file sciadv.adg5391_sm.pdf]

Science Advances
AAAS

Supplementary Materials for

IRF1 regulates self-renewal and stress-responsiveness to support hematopoietic stem cell maintenance

Alexandra J.S. Rundberg Nilsson *et al.*

Corresponding author: Alexandra J.S. Rundberg Nilsson, alexandra.rundberg_nilsson@med.lu.se

Sci. Adv. **9**, eadg5391 (2023)
DOI: 10.1126/sciadv.adg5391

The PDF file includes:

Figs. S1 to S8
Tables S4, S5, S6, S7 and S9
Legends for tables S1, S2, S3 and S8

Other Supplementary Material for this manuscript includes the following:

Tables S1, S2, S3 and S8

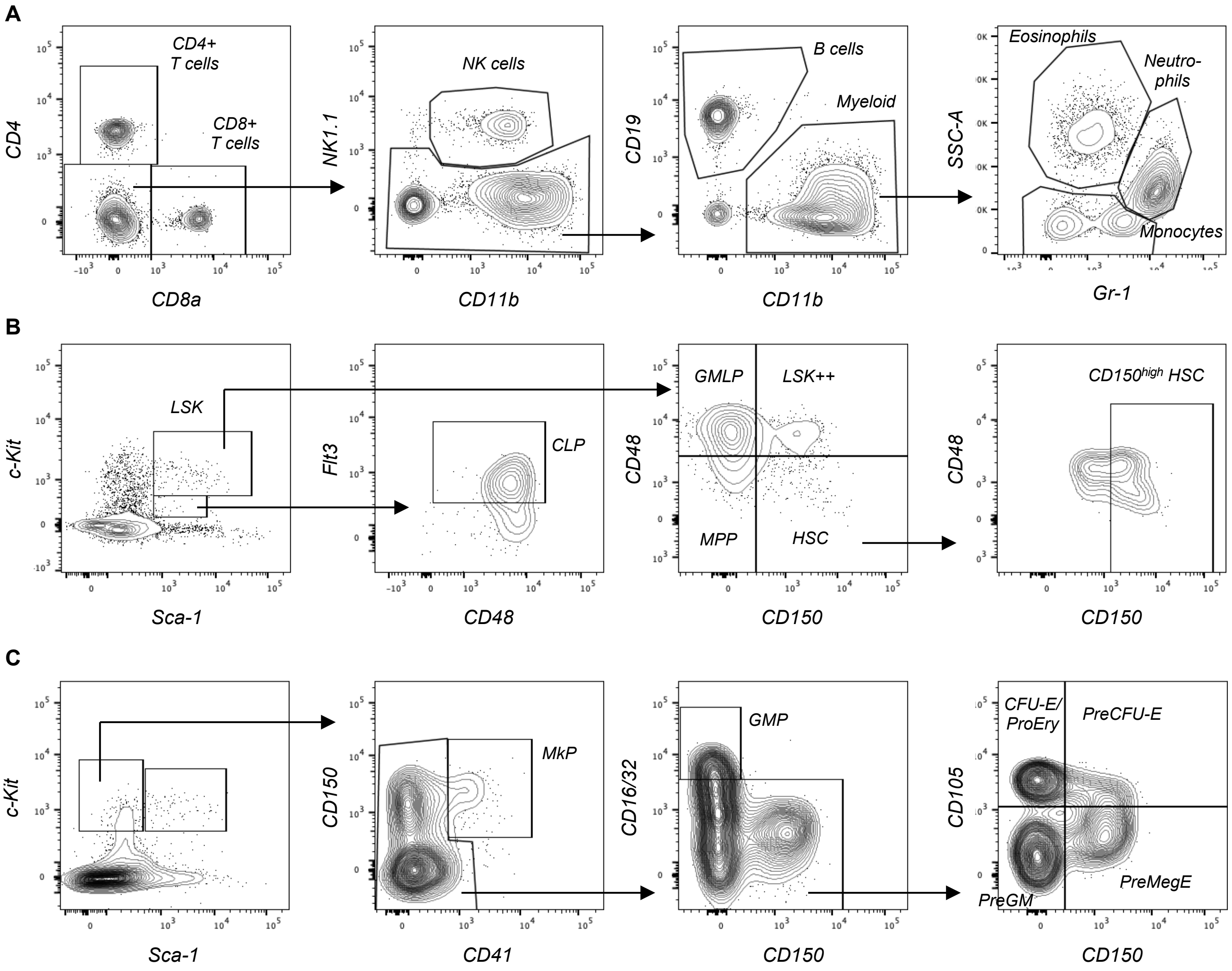

Fig. S1. Representative peripheral blood and bone marrow gating strategies. (A) Representative gating strategy for PB subsets. Pre-gated on singlets, viability, and scatter. Gating strategy for **(B)** BM HSPCs and CLPs, and **(C)** BM myeloid, erythroid and megakaryocytic precursors. Pre-gated on singlets, viability, scatter, and lineage negative.

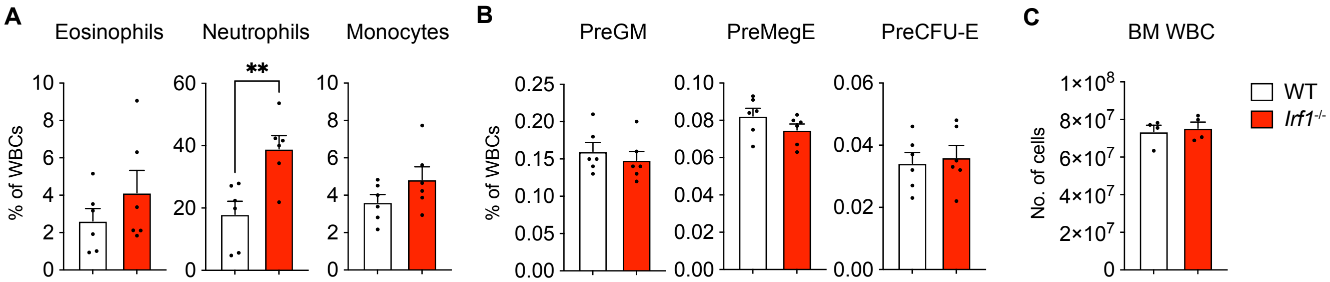

Fig. S2. *Irf1*$^{-/-}$ mice show alterations in hematopoietic compartments. (A) Myeloid subpopulation frequencies within PB WBCs. **(B)** Intermediate progenitor population frequencies within BM WBCs. WT n=6, *Irf1*$^{-/-}$ n=6. **(C)** BM WBC counts. WT n=4, *Irf1*$^{-/-}$ n=4. Error bars represent +SEM. P values were calculated by two-tailed student's t-test. *p < 0.05, **p < 0.01, ***p < 0.001, ****p < 0.0001.

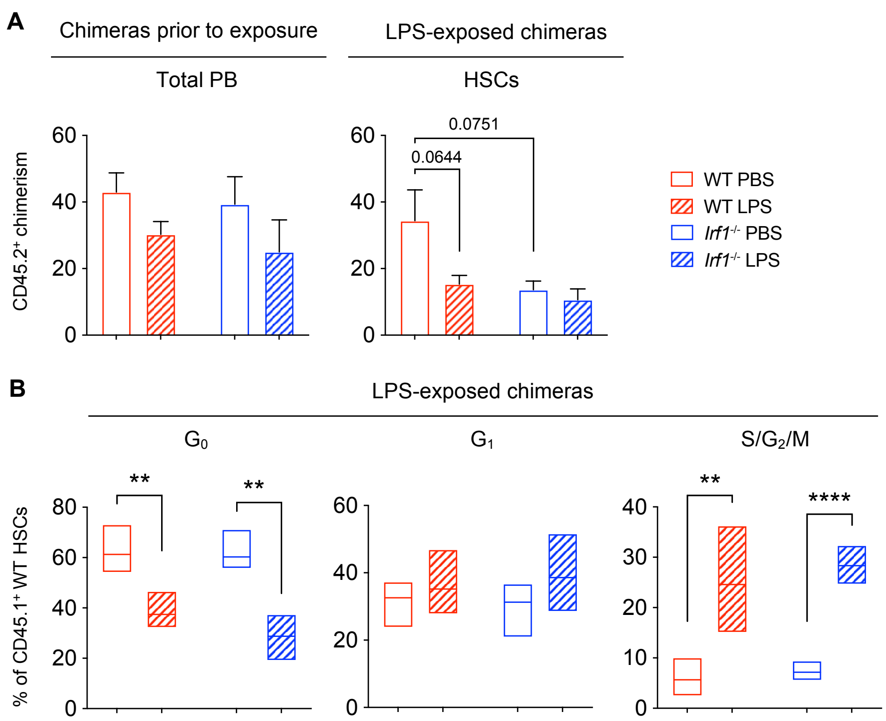

Fig. S3. Partial presence of *Irf1*$^{-/-}$ hematopoietic cells does not alter LPS-induced proliferation responses of WT HSCs. (A) CD45.2^{+} chimerism levels in PB (pre-treatment, left), and in HSCs (post treatment, right) at 12 weeks after transplantation. Error bars represent +SEM. **(B)** Endogenous and competitor WT donor CD45.1^{+} HSC cell cycle distribution in chimeric mice. Color/pattern marking indicates treatment and CD45.2^{+} donor BM. Box plots show floating bars (min to max) with line a mean. WT PBS n=3, WT LPS n=5, *Irf1*$^{-/-}$ PBS n=4, *Irf1*$^{-/-}$ LPS n=3. P values were calculated by two-tailed student's t-test. *$p < 0.05$, **$p < 0.01$, ***$p < 0.001$, ****$p < 0.0001$.

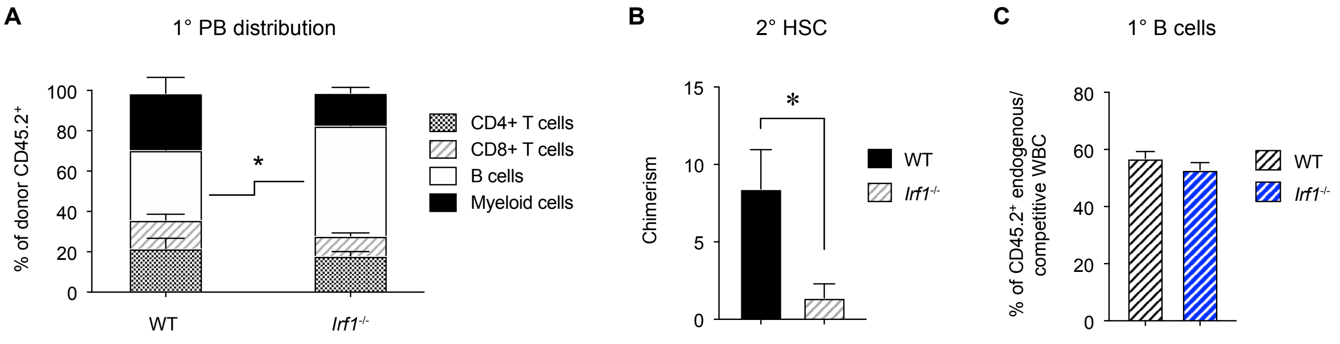

Fig. S4. *Irf1*$^{-/-}$ HSCs exhibit B cell biased mature peripheral blood output and impaired serial reconstitution capacity. (A) CD45.2^{+} PB distribution levels at 20 weeks post 1° competitive HSC transplantation. WT n=6, *Irf1*$^{-/-}$ n=6. **(B)** HSC chimerism levels at 23 weeks post serial HSC transplantation. WT n=6, *Irf1*$^{-/-}$ n=6. **(C)** Endogenous/competitor CD45.2^{+} B cell distribution in WT and *Irf1*$^{-/-}$ hosts. WT n=8, *Irf1*$^{-/-}$ n=6. Error bars represent +SEM. P values were calculated by two-tailed student's t-test. *p < 0.05, **p < 0.01, ***p < 0.001, ****p < 0.0001.

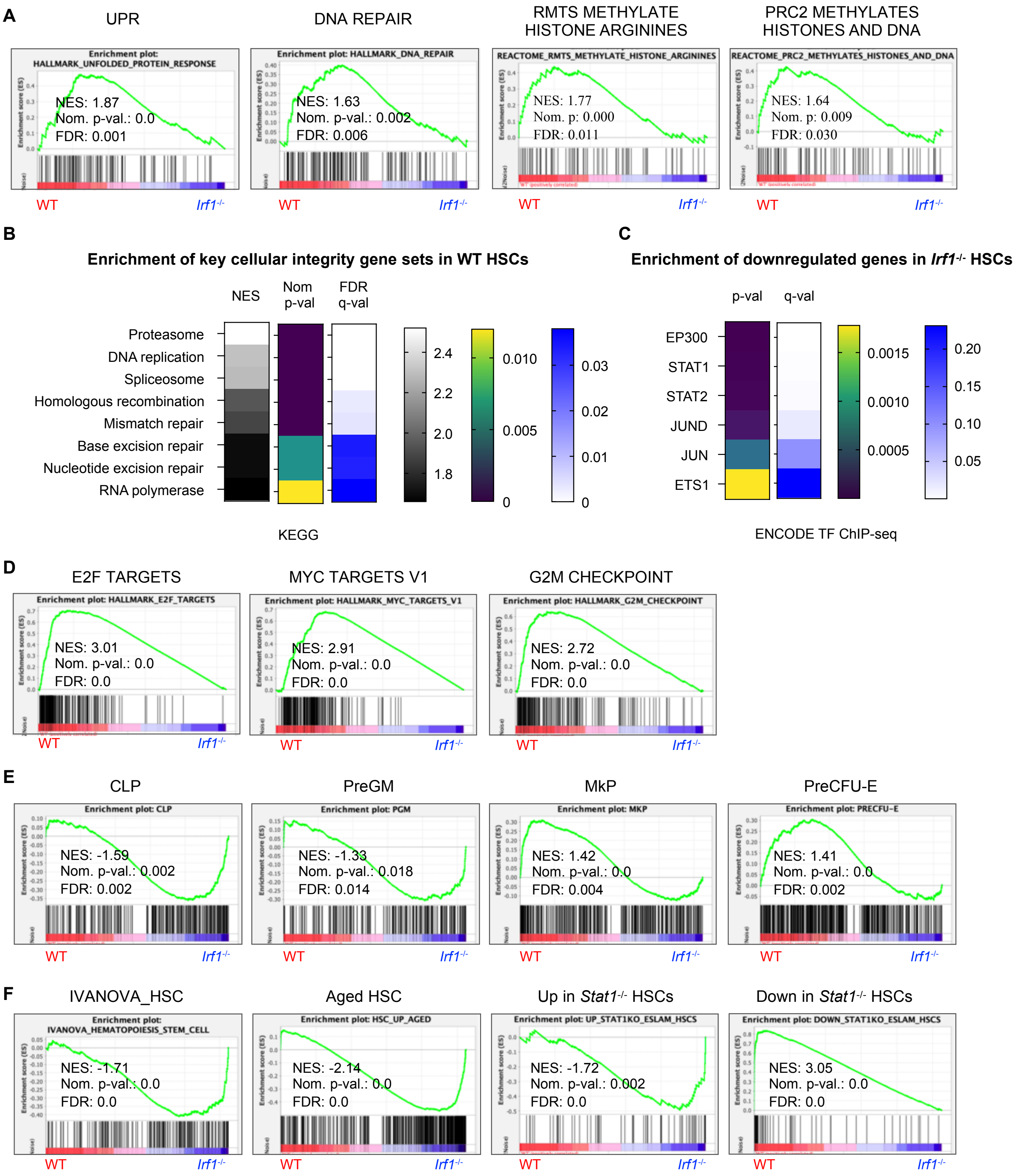

Fig. S5. Altered gene expression landscape in *Irf1*$^{-/-}$ HSCs.

GSEA of **(A)** unfolded protein response (UPR), DNA repair, and histone modification gene sets between WT and *Irf1*$^{-/-}$ HSCs. **(B)** GSEA of selected KEGG pathways between WT and *Irf1*$^{-/-}$ HSCs. **(C)** Enrichment analysis of downregulated genes in *Irf1*$^{-/-}$ HSCs to ENCODE TF ChIP-seq gene sets. GSEA of **(D)** proliferation-, **(E)** lineage-, and **(F)** HSC function-associated gene sets between WT and *Irf1*$^{-/-}$ HSCs.

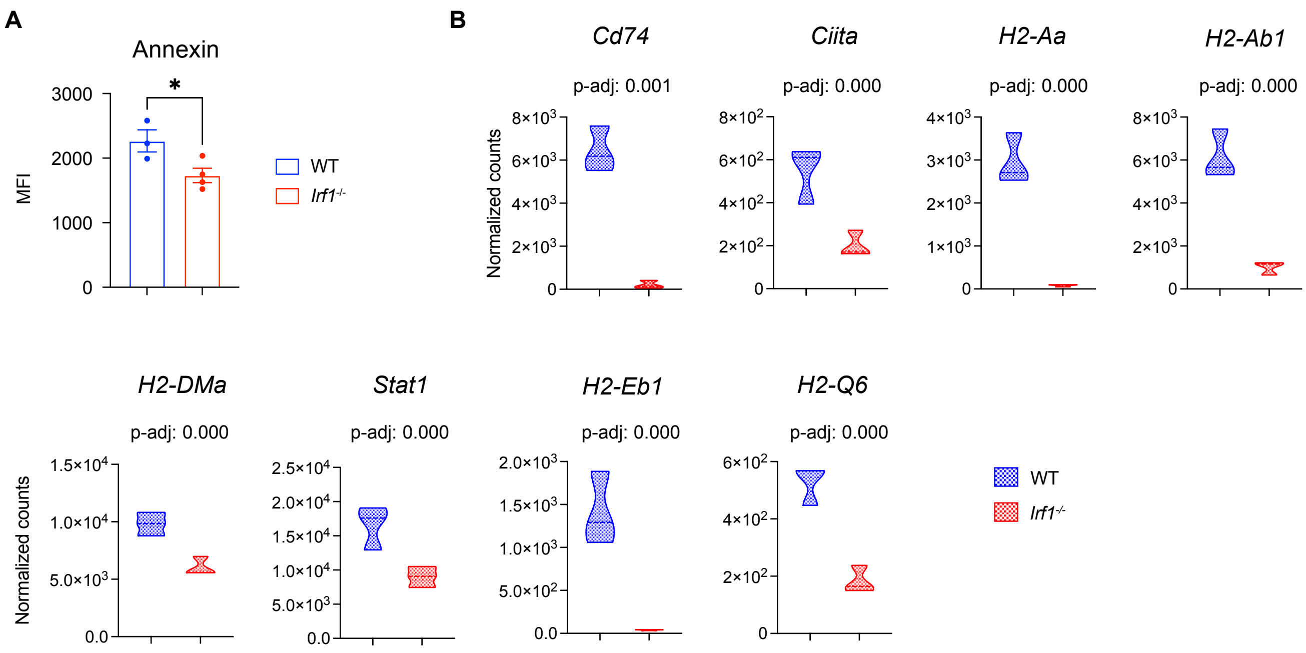

Fig. S6. Reduced apoptosis and antigen presentation in *Irf1*$^{-/-}$ HSCs.
(A) Quantification of Annexin V levels in WT and *Irf1*$^{-/-}$ HSCs. WT n=3, *Irf1*$^{-/-}$ n=4. P values were calculated by two-tailed student's t-test. *$p < 0.05$, **$p < 0.01$, ***$p < 0.001$, ****$p < 0.0001$. **(B)** Truncated violin plots displaying expression levels of selected genes associated with antigen presentation via MHC class II in WT and *Irf1*$^{-/-}$ HSCs.

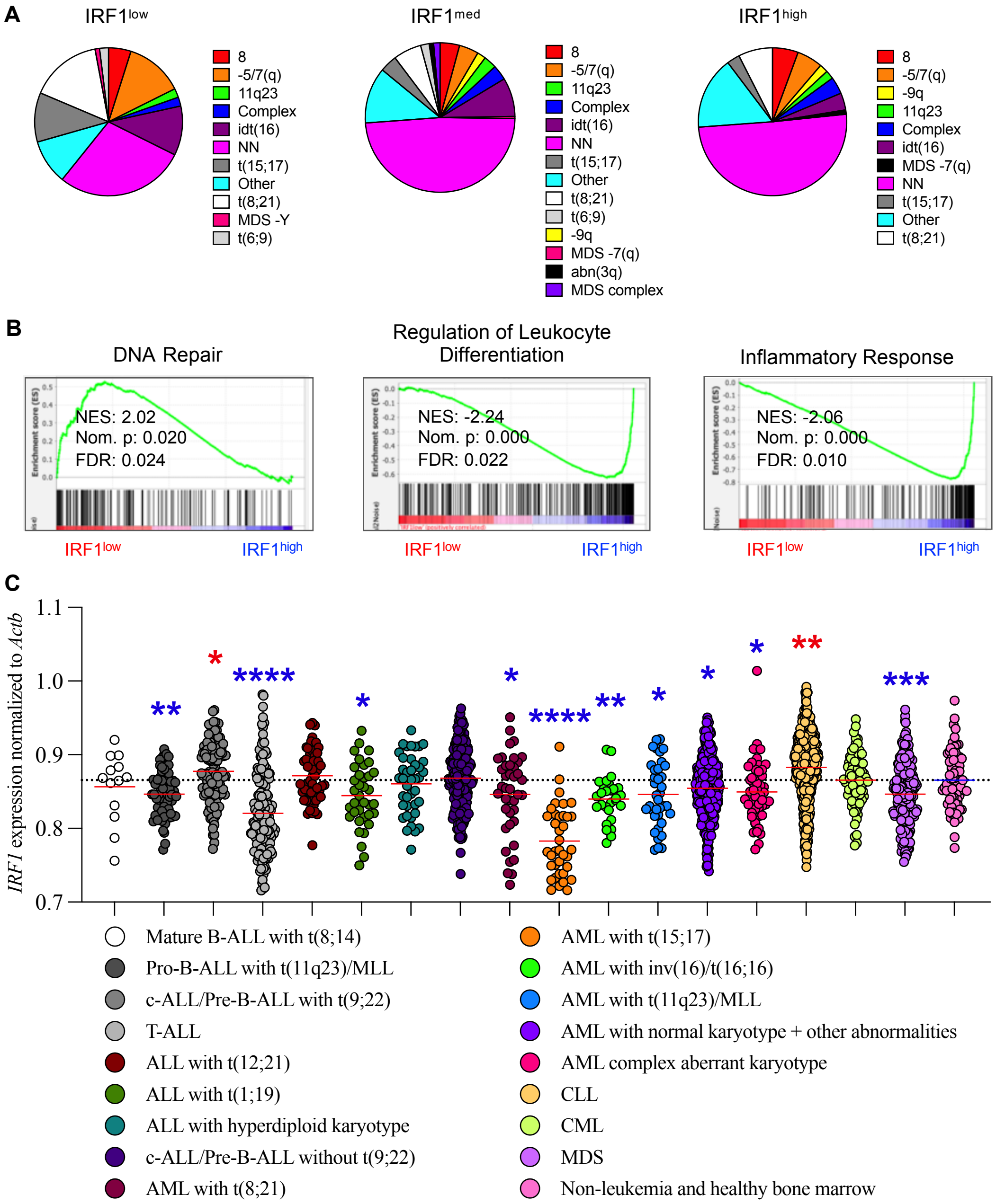

Fig. S7. *IRF1*-based AML patient stratification identifies distinct subgroups.
(A) Karyotype distribution within the three *IRF1* expression level groups. 8 = trisomy 8, -5/7(q), -Y, -9q, -7(q) = loss of chromosomal segments, 11q23, t(15;17, t(8;21), t/6;9) = translocations, idt(16) = inversion, NN = normal karyotype, abn = abnormal, MDS = myelodysplastic syndrome. IRF1^{low} n=102, IRF1^{med} n=214, IRF1^{high} n=107. **(B)** GSEA between IRF1^{low} and IRF1^{high} patient samples for DNA repair, regulation of leukocyte differentiation, and inflammatory response gene sets. IRF1^{low} n=135, IRF1^{med} n=268, IRF1^{high} n=134. **(C)** *IRF1* gene expression in various leukemia subclasses (GSE13159). Blue asterisks indicate significant downregulation compared to normal BM, and red asterisks indicate significant upregulation. Dotted horizontal line depicts mean level in healthy BM. Mature B-ALL with t(8;14) n=13, Pro-B-ALL with t(11q23)/MLL n=70, c-ALL/Pre-B-B-ALL with t(9;22) n=122, T-ALL n=174, ALL with t(12;21) n=58, ALL

with t(1;19) n=36, ALL with hyperdiploid karyotype n=40, c-ALL/Pre-B-ALL without t(9;22) n=237, AML with t(8;21) n= 40, AML with t(15;17) n=37, AML with inv(16)/t(16;16) n=28, AML with t(11q23)/MLL n=38, AML with normal karyotype + other abnormalities n=351, AML with complex aberrant karyotype n=48, CLL n=448, CML n=76, MDS n=206, Non-leukemia and healthy bone marrow n=74. P values were calculated by two-tailed student's t-test. *p < 0.05, **p < 0.01, ***p < 0.001, ****p < 0.0001.

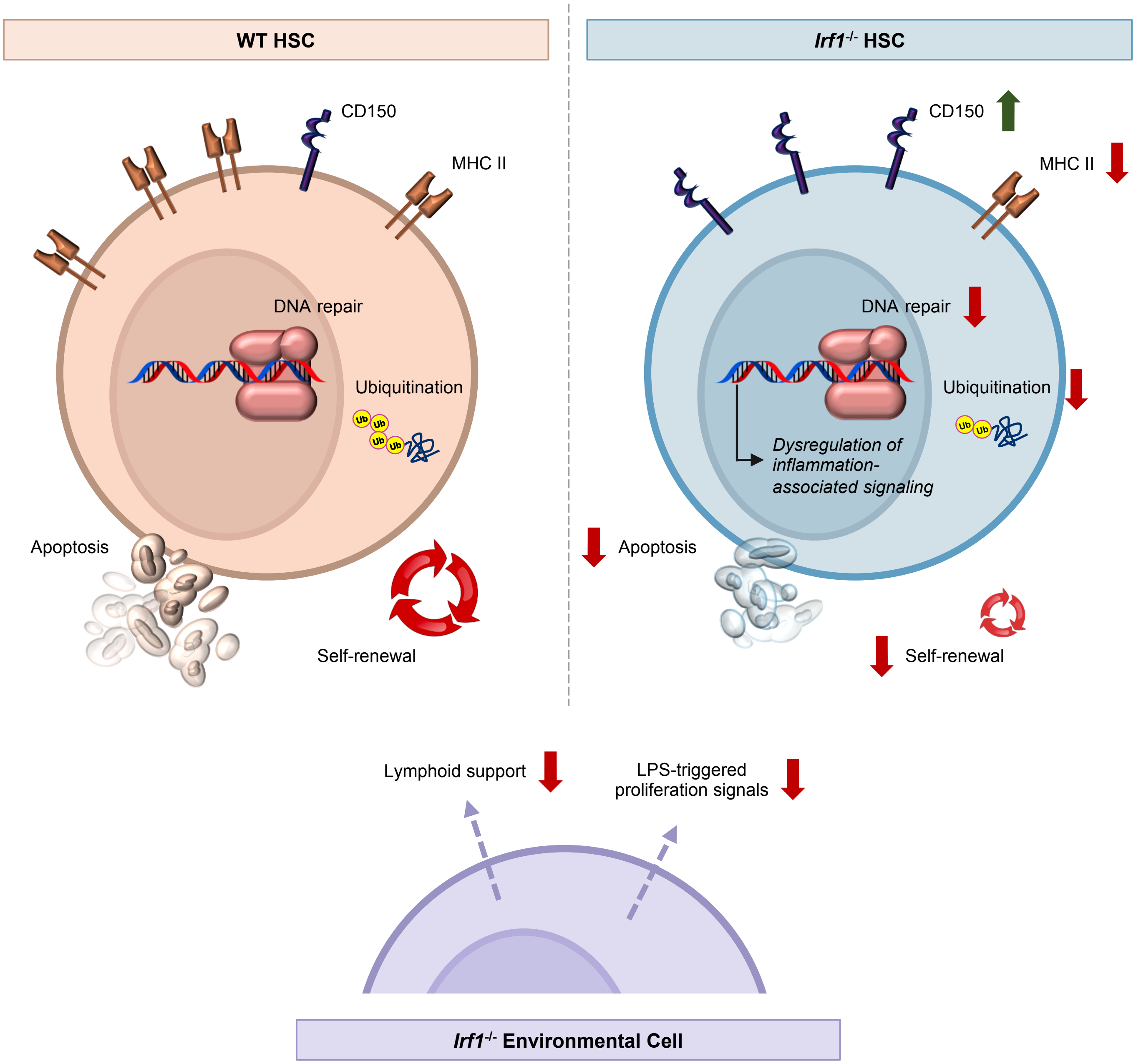

Fig. S8. IRF1 regulates key HSC processes. IRF1 is a pivotal HSC regulator, controlling long-term self-renewal, apoptosis, DNA repair, protein degradation and inflammatory signaling. IRF1 also regulates the expression of fundamental surface markers, including CD150 and MHC class II. Moreover, IRF1 controls lymphoid development and HSC proliferation through extrinsic signaling.

Table S4. Significantly enriched MSigDB Hallmark gene sets among downregulated genes in *Irf1*$^{-/-}$ HSCs identified with Enrichr.

Term	Overlap	P-val	Adj. P-val	Odds Ratio	Combined Score	Genes
Interferon Gamma Response	26/200	2,78E-26	9,74E-25	2,72E+01	1,60E+03	CD86;CIITA;DDX60;IFIT3;PSMB10;IFIT2;IL18BP;CASP1;ITGB7;B2M;GBP4;ZBP1;GBP6;BATF2;CD74;STAT1;MX1;TRAFD1;TAP1;PSMB8;PSMB9;ISG20;IFI27;IL7;OAS3;PSME2
Interferon Alpha Response	18/97	2,45E-21	4,29E-20	3,89E+01	1,84E+03	BATF2;CD74;MX1;TAP1;TRAFD1;DDX60;IFIT3;PSMB8;IFIT2;PSMB9;ISG20;IFI27;IL7;CASP1;PSME2;GBP2;B2M;GBP4
Allograft Rejection	13/200	9,27E-10	1,08E-08	1,13E+01	2,35E+02	CD86;ACHE;CD74;STAT1;F2R;LY75;TAP1;PSMB10;IL7;IL12RB1;GBP2;B2M;PF4
IL-6/JAK/STAT3 Signaling	4/87	2,79E-03	2,44E-02	7,33E+00	4,31E+01	STAT1;IL7;IL12RB1;PF4
Apoptosis	5/161	4,61E-03	3,23E-02	4,90E+00	2,63E+01	ISG20;TOP2A;F2R;CASP1;TAP1

Table S5. Significantly enriched KEGG pathway gene sets among downregulated genes in *Irf1*$^{-/-}$ HSCs identified with Enrichr.

Term	Overlap	P-val	Adj. P-val	Odds Ratio	Combined Score	Genes
Proteasome	4/46	2,53E-04	9,94E-03	1,45E+01	1,20E+02	PSME2;PSMB10;PSMB8;PSMB9
Antigen processing and presentation	5/78	1,79E-04	9,94E-03	1,05E+01	9,07E+01	CD74;CIITA;TAP1;PSME2;B2M
NOD-like receptor signaling pathway	7/181	2,21E-04	9,94E-03	6,24E+00	5,25E+01	CASP12;STAT1;OAS3;CASP1;GBP2;GBP4;GBP3

Table S6. Significantly enriched ENCODE TF ChIP gene sets among downregulated genes in *Irf1*$^{-/-}$ HSCs identified with WebGestalt.

Term	p-value	q-value
EP300 CH12.LX mm9	1,80E-09	1,41E-06
STAT1 K562 hg19	3,67E-06	1,43E-03
STAT2 K562 hg19	1,47E-05	3,83E-03
JUND CH12.LX mm9	9,54E-05	1,86E-02
JUN CH12.LX mm9	6,39E-04	9,96E-02
ETS1 MEL cell line mm9	1,78E-03	2,31E-01
TAL1 MEL cell line mm9	2,38E-03	2,66E-01
TAL1 megakaryocyte mm9	5,61E-03	5,47E-01
RELA GM12878 hg19	6,55E-03	5,67E-01
STAT1 HeLa-S3 hg19	1,09E-02	8,54E-01
ETS1 CH12.LX mm9	1,36E-02	9,67E-01
PRDM1 HeLa-S3 hg19	2,37E-02	1,00E+00
EP300 MEL cell line mm9	2,53E-02	1,00E+00
CHD1 CH12.LX mm9	4,47E-02	1,00E+00

Table S7. Significantly enriched MSigDB Halllmark pathways among upregulated genes in *Irf1*$^{-/-}$ HSCs identified with Enrichr.

Term	Over-lap	P-val	Adj. P-val	Odds Ratio	Combined Score	Genes
IL-6/JAK/STAT3 Signaling	5/87	8,58E-04	1,14E-02	7,34E+00	5,18E+01	SOCS3;IL1R1;CSF2RB;CD14;IL18R1
Coagulation	6/138	1,14E-03	1,14E-02	5,49E+00	3,72E+01	DPP4;MMP14;SPARC;ACOX2;MMP2;CTSH
TNF-alpha Signaling via NF-kB	7/200	1,59E-03	1,14E-02	4,40E+00	2,83E+01	EFNA1;SOCS3;EGR1;CEBPD;NFIL3;BCL3;FOSL2
Epithelial Mesenchymal Transition	7/200	1,59E-03	1,14E-02	4,40E+00	2,83E+01	EFEMP2;MMP14;SPARC;LGALS1;ITGB5;MMP2;TGFBI
Inflammatory Response	7/200	1,59E-03	1,14E-02	4,40E+00	2,83E+01	IFITM1;MMP14;IL18RAP;IL1R1;IL10RA;CD14;IL18R1
Hypoxia	6/200	7,15E-03	3,68E-02	3,73E+00	1,84E+01	EFNA1;NFIL3;TGFBI;VLDLR;PPARGC1A;FOSL2
Xenobiotic Metabolism	6/200	7,15E-03	3,68E-02	3,73E+00	1,84E+01	CYP27A1;ACOX2;IL1R1;PDK4;RAP1GAP;PTGES

Table S9. Key resources table containing detailed information about antibodies, reagents, data, and instruments used in the study.

REAGENT or RESOURCE	SOURCE	IDENTIFIER
**Antibodies**		
Biotin anti-mouse/human CD45R/B220 (RA3-6B2)	BioLegend	Cat: 103204
Biotin anti-mouse CD4 (GK1.5)	BioLegend	Cat: 100404
Biotin anti-mouse CD8a (53-6.7)	BioLegend	Cat: 100704
Biotin anti-mouse/human CD11b (M1/70)	BioLegend	Cat: 101204
Biotin anti-mouse Ly-6G/Ly6C (Gr-1) (RB6-8C5)	BioLegend	Cat: 108404
Biotin anti-mouse TER-119/Erythroid Cells (TER-119)	BioLegend	Cat: 116204
PE/Cy5 anti-mouse/human CD45R/B220 (RA3-6B2)	BioLegend	Cat: 103209
PE/Cy5 anti-mouse/human CD11b (M1/70)	BioLegend	Cat: 101209
PE/Cy5 anti-mouse TER-119/Erythroid Cells (TER-119)	BioLegend	Cat: 116209
APC/Cy7 anti-mouse CD4 (RM4-5)	BioLegend	Cat: 100526
PE/Cy5 anti-mouse CD8a (53-6.7)	BioLegend	Cat: 100709
APC anti-mouse human CD11b (M1/70)	BioLegend	Cat: 101212
PE/Cy7 anti-mouse CD19 (6D5)	BioLegend	Cat: 115520
PE anti-mouse CD45.1 (A20)	BioLegend	Cat: 110708
FITC anti-mouse CD45.2 (104)	BioLegend	Cat: 109806
APC-eFluor780 anti-mouse CD117 (c-Kit) (2B8)	Invitrogen	Ref: 47-1171-82
Pacific Blue anti-mouse Ly-6A/E (Sca-1) (E13-16.7)	BioLegend	Cat: 122520
NK.1. Brilliant Violet 421	BioLegend	Cat: 108722
Alexa Fluor 700 anti-mouse CD48 (HM48-1)	BioLegend	Cat: 103426
PE/Cy7 anti-mouse CD48 (HM48-1)	BioLegend	Cat: 103424
APC anti-mouse CD150 (SLAM) (TC15-12F12.2)	BioLegend	Cat: 115910
PE anti-mouse CD135 (FLT3) (A2F10))	BioLegend	Cat: 135306
PE/Cy7 anti-mouse CD105 (MJ7/18)	BioLegend	Cat: 120410
PerCP-eFluor710 anti-mouse CD41 (eBioMWReg30)	Invitrogen	Ref: 46-0411-82
PE anti-mouse CD41 (MWReg30)	BioLegend	Cat: 133906
Alexa Fluor 700 anti-mouse CD16/32 (93)	Invitrogen	Ref: 56-0161-82
FITC anti-mouse Ki67	BD Biosciences	Cat: 556026
PE anti-mouse Ki67	BD Biosciences	Cat: 556027
Brilliant Violet 510 Streptavidin	BioLegend	Cat: 405234
FITC anti-mouse MHC II	Miltenyi Biotec	130-123-666
Multi ubiquitin mAb (D071-3)	MBL International Corporation	D071-3
Alexa Fluor 488 goat anti-mouse IgG (H+L) Cross-Adsorbed ReadyProbes^{TM} Secondary Antibody	Invitrogen	Cat: R37120
**Chemicals, peptides, and recombinant proteins**		
7-Aminoactinomycin D (7-AAD)	Invitrogen	Cat: A1310
Propidium Iodide	Molecular Probes	Cat: P3566
Heparin solution	Stem Cell Technologies	Cat: 07980
Lipopolysaccharide (LPS)	Sigma Aldrich	Product No: L4005
**Critical commercial assays**		

BD cytofix/cytoperm fixation/permeabilization solution kit	BD Bioscience	Cat: BD 554714
BD FITC Mouse Anti-Ki-67 Set	BD Biosciences	Cat: 556026
SMART-Seq® v4 Ultra® Low Input RNA Kit	Takara Bio USA, Inc.	Cat. Nos. 634888, 634889, 634890, 634891, 634892, 634893, 634894 (091817)
Single Cell RNA Purification Kit	Norgen Biotek Corp.	Cat: 52800
Illumina Nextera XT kit	Illumina	4456740
BD Annexin V: FITC Apoptosis Detection Kit I	BD Pharmingen™	BD 556547
**Deposited data**		
RNA-seq data	This manuscript	GSE220263
**Publicly available data**		
Expression profiling by array: AML samples		GSE6891
Expression profiling by array: Microarray Innovations in Leukemia (MILE) study		GSE13159
**Experimental models: Organisms/strains**		
Mouse: C57Bl/6JRj	In-house breeding	
Mouse: B6.SJL (B6.SJL-*Ptprc*^{a}*Pepc*^{b}/BoyCrCrl)	In-house breeding	Charles River, Strain Code: 564
Mouse: IRF1 KO (B6.129S2-*Irf1*^{tm1Mak}/J)	In-house breeding	Jackson Laboratory, stock number 002762
**Software and algorithms**		
FlowJo	BD, Treestar	v. 10
GraphPad Prism	Dotmatics	v. 9
Gene Set Enrichment Analysis (GSEA) software	Broad institute	http://software.broadinstitute.org/gsea/index.jsp
Enrichr		https://maayanlab.cloud/Enrichr/
WEB-based Gene SeT AnaLysis Toolkit (WebGestalt)		https://www.webgestalt.org/
RNA Galaxy workbench 2.0	RNA Bioinformatics Center (RBC)	https://usegalaxy.eu/
TapeStation Analysis Software 3.2	Agilent Technologies, Inc.	
Cell Radar		https://karlssong.github.io/cellradar/
**Other**		
BD Aria III	Becton Dickinson	
BC CyAn ADP	Beckman Coulter	
BD LSR X-20	Becton Dickinson	
NovaSeq S4	Illumina	

Supplemental auxiliary files:

Table S1. Significantly downregulated genes in *Irf1*$^{-/-}$ HSCs *versus* WT HSCs.

Table S2. Significantly upregulated genes in *Irf1*$^{-/-}$ HSCs *versus* WT HSCs.

Table S3. Top 50 predicted upstream regulators for downregulated genes in *Irf1*$^{-/-}$ HSCs.

Table S8. Cell type-specific gene sets.
